# Supplementary material for: Plant beta-diversity across biomes captured by imaging spectroscopy
Source: Nat Commun. 2022 May 19;13:2767. doi: 10.1038/s41467-022-30369-6 (PMC9120498; doi:10.1038/s41467-022-30369-6)
Supplement: Supplementary file 1 — Supplementary Information file [file 41467_2022_30369_MOESM1_ESM.pdf]

Supplementary Information

Plant beta-diversity across biomes captured by imaging spectroscopy

Schweiger, A.K. & Laliberté, E.

Content

- **Supplementary Fig. 1:** Synthetic landscape consisting of 24 20 m × 20 m plant communities (research plots) at the University of Kansas Field Station.
- **Supplementary Fig. 2:** Pairwise spectral distance among plots increases with their taxonomic distance.
- **Supplementary Fig. 3:** Relationships between plant diversity and spectral diversity at the alpha-scale per site.
- **Supplementary Fig. 4:** Scaling plant inventories to vegetation cover as seen from above.
- **Supplementary Table 1:** NEON sites used in this study, site characteristics.
- **Supplementary Table 2:** Linear regression models between taxonomic and spectral distances among plots per site.
- **Supplementary Table 3:** Covariance between plot-wise ordinations of plant species inventories and mean spectra per site.
- **Supplementary Table 4:** Best mixed effects model predicting plant species richness.
- **Supplementary Table 5:** Best mixed effects model predicting the Shannon index of plant diversity.
- **Supplementary Table 6:** Best mixed effects model predicting plant phylogenetic diversity.
- **Supplementary References**

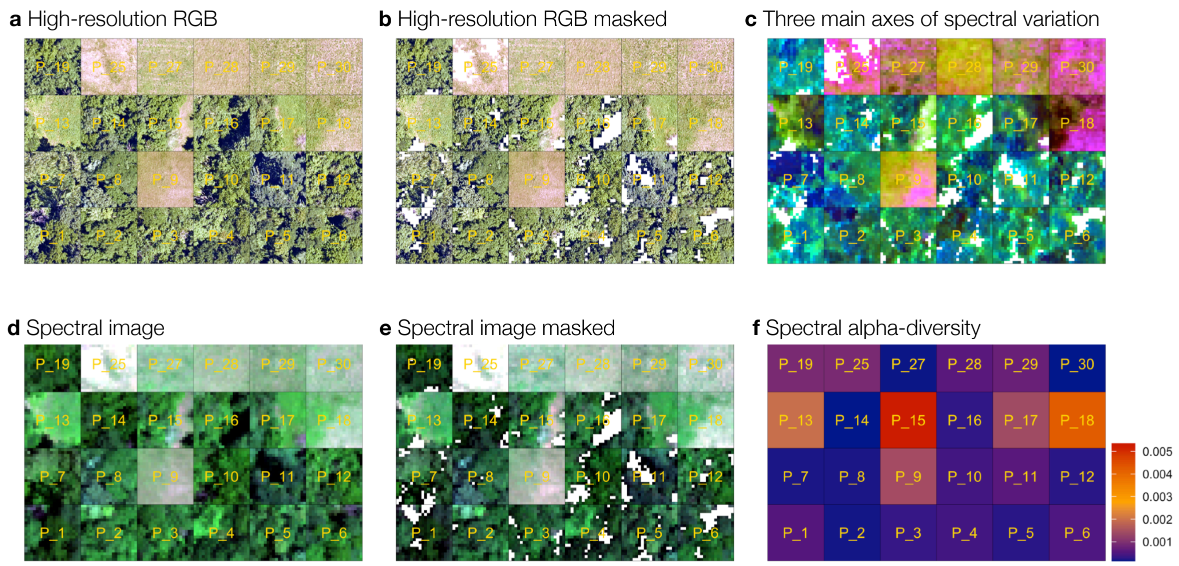

Supplementary Fig. 1: Synthetic landscape consisting of 24 20 m x 20 m plant communities (research plots) at the University of Kansas Field Station. **a** High-resolution true color images (RGB) with and **b** without masked pixels, **c** the three main axes of spectral variation, **d** spectral RGB images with and **e** without masked pixels, **f** spectral alpha-diversity, colors represent a gradient from low (blue) to high (red) spectral alpha-diversity values.

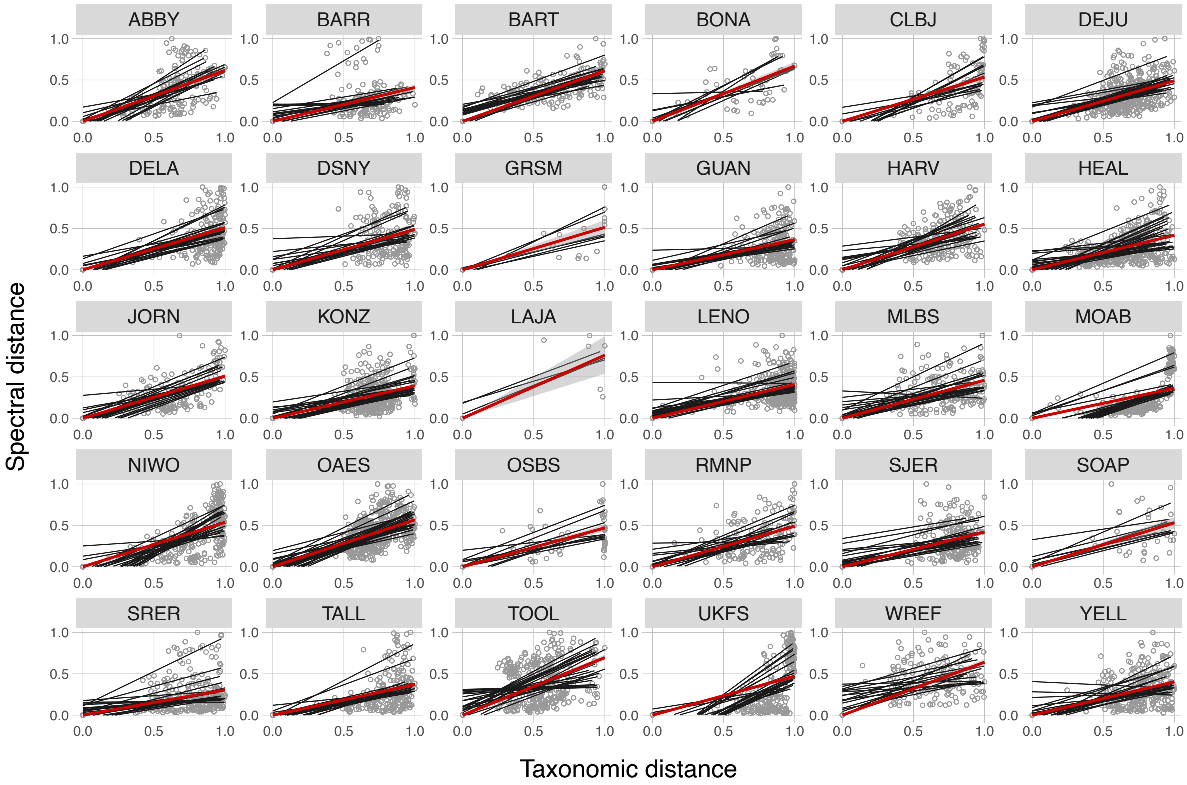

Supplementary Fig. 2: Pairwise spectral distance among plots increases with their taxonomic distance. The red line is the mean fitted regression line for all plots per NEON site; bands indicate 95% confidence intervals. Grey lines represent fitted linear regression models predicting for each plot per site spectral distance from taxonomic distance; circles represent plots. For site abbreviations see Supplementary Table 1, for statistics see Supplementary Table 2.

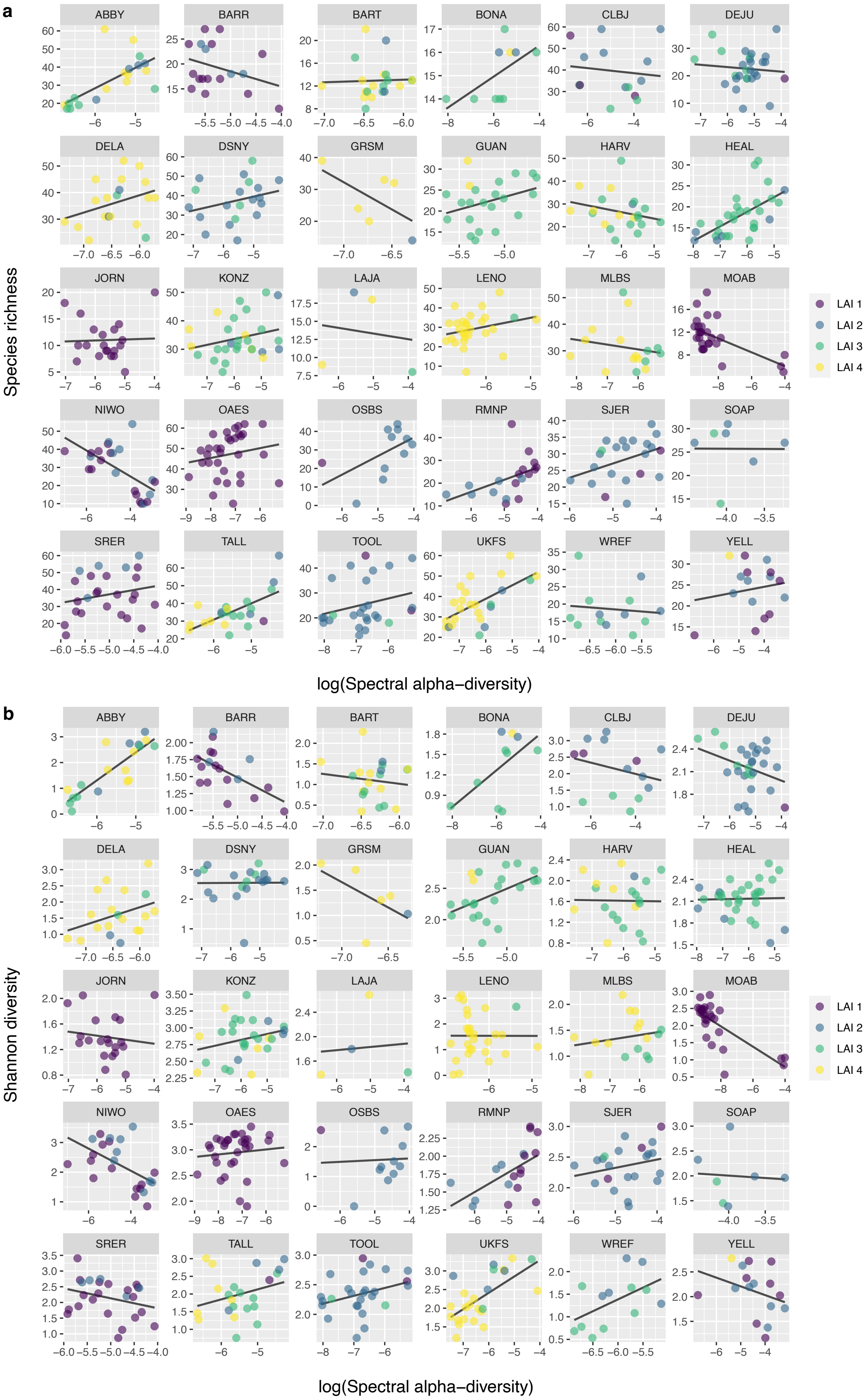
a
ABBY
BARR
BART
BONA
CLBJ
DEJU
DELA
DSNY
GRSM
GUAN
HARV
HEAL
JORN
KONZ
LAJA
LENO
MLBS
MOAB
NIWO
OAES
OSBS
RMNP
SJER
SOAP
SREF
TALL
TOOL
UKFS
WREF
YELL
Species richness
log(Spectral alpha−diversity)
LAI 1
LAI 2
LAI 3
LAI 4
b
ABBY
BARR
BART
BONA
CLBJ
DEJU
DELA
DSNY
GRSM
GUAN
HARV
HEAL
JORN
KONZ
LAJA
LENO
MLBS
MOAB
NIWO
OAES
OSBS
RMNP
SJER
SOAP
SRER
TALL
TOOL
UKFS
WREF
YELL
Shannon diversity
log(Spectral alpha−diversity)
LAI 1
LAI 2
LAI 3
LAI 4

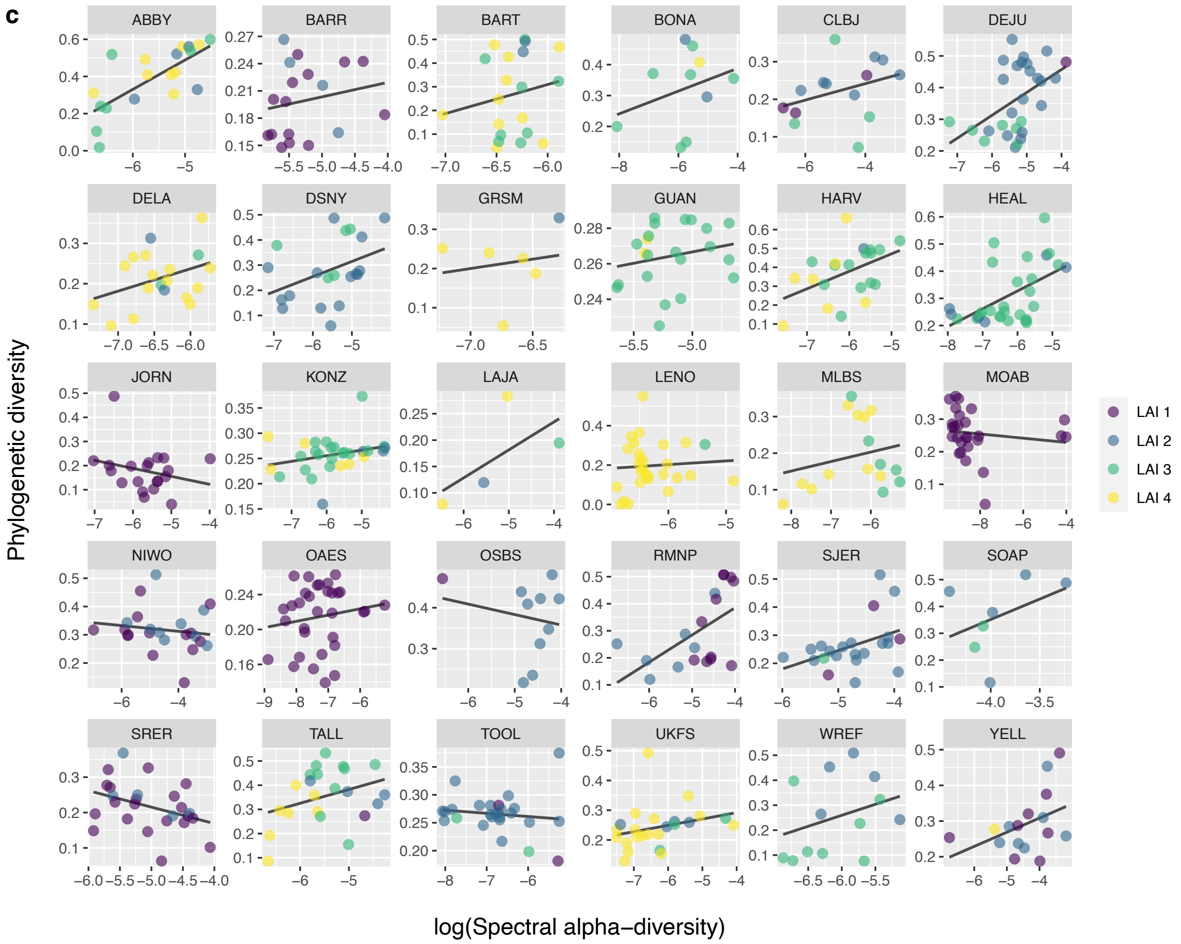

Supplementary Fig. 3: Relationships between plant diversity and spectral diversity at the alpha-scale per site. Relationships between spectral alpha-diversity and measured **a** plant species richness, **b** Shannon index and **c** phylogenetic diversity. As phylogenetic diversity measure, we used phylogenetic species evenness (PSE). Colors represent four classes of the leaf area index (LAI) ranging from open to closed canopies: LAI 1 = [0.13, 0.634], LAI 2 = (0.634, 1.183], LAI 3 = (1.183, 1.84], LAI 4 = (1.84, 3.80], NS: $P > 0.1$); the black line shows the overall relationship per site, for site abbreviations see Supplementary Table 1.

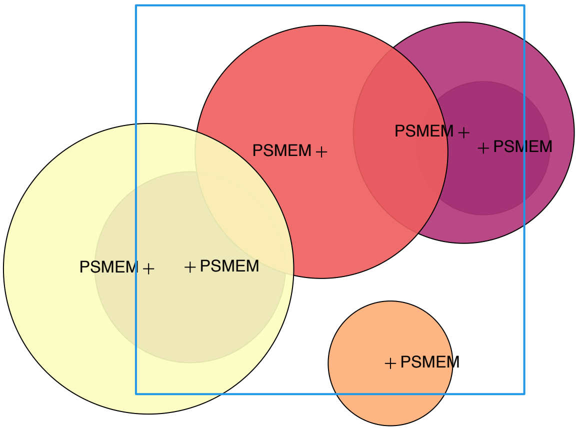

Supplementary Fig. 4: Scaling plant inventories to vegetation cover as seen from above. Similar to this example of plot 1 (delineated by the blue square) at the Abby Road site, we mapped all trees per plot based on their location, height and crown diameter. We calculated the area within the plot covered by each tree species, and scaled percent cover of herbaceous and understory plants to the area within the plot not covered by trees (white area within the blue square).

Supplementary Table 1: NEON sites used in this study.

Site ID	Site Name	State	Elevation	MAP	MAT	Domain Name	Latitude	Longitude	LAI
ABBY	Abby Road	WA	363 m	2530 mm	8C/46.4F	Pacific Northwest	45.76243	-122.33033	1.78
BARR	Barrow Environmental Observatory	AK	6 m	110 mm	-12C/10.4F	Tundra	71.28241	-156.61936	0.52
BART	Bartlett Experimental Forest	NH	232 m	1056 mm	8C/46.4F	Northeast	44.06388	-71.28731	1.90
BONA	Caribou-Poker Creeks Research Watershed	AK	263 m	399 mm	-1C/30.2F	Taiga	65.15401	-147.50258	1.38
CLBJ	LBJ National Grassland	TX	259 m	840 mm	18C/64.4F	Southern Plains	33.40123	-97.57000	0.94
DEJU	Delta Junction	AK	529 m	300 mm	-2C/28.4F	Taiga	63.88112	-145.75136	1.07
DELA	Dead Lake	AL	22 m	1290 mm	18C/64.4F	Ozarks Complex	32.54172	-87.80389	2.39
DSNY	Disney Wilderness Preserve	FL	15 m	1150 mm	22C/71.6F	Southeast	28.12504	-81.43620	1.10
GRSM	Great Smoky Mountains National Park, Twin Creeks	TN	579 m	1396 mm	13C/55.4F	Appalachians & Cumberland Plateau	35.68896	-83.50195	2.72
GUAN	Guanica Forest	PR	143 m	1168 mm	25C/77F	Atlantic Neotropical	17.96955	-66.86870	1.55
HARV	Harvard Forest	MA	351 m	967 mm	8C/46.4F	Northeast	42.53690	-72.17266	1.70
HEAL	Healy	AK	705 m	320 mm	-4C/24.8F	Taiga	63.87569	-149.21334	1.31
JORN	Jornada LTER	NM	1329 m	173 mm	17C/62.6F	Desert Southwest	32.59068	-106.84254	0.20
KONZ	Konza Prairie Biological Station	KS	381 m	860 mm	12C/53.6F	Prairie Peninsula	39.10077	-96.56309	1.63
LAJA	Lajas Experimental Station	PR	24 m	1168 mm	25C/77F	Atlantic Neotropical	18.02125	-67.07690	1.55
LENO	Lenoir Landing	AL	20 m	1429 mm	18C/64.4F	Ozarks Complex	31.85388	-88.16122	2.60
MLBS	Mountain Lake Biological Station	VA	1126 m	1030 mm	13C/55.4F	Appalachians & Cumberland Plateau	37.37828	-80.52484	2.07
MOAB	Moab	UT	1767 m	200 mm	11C/51.8F	S-Rockies & Colorado Plateau	38.24833	-109.38827	0.27
NIWO	Niwot Ridge Mountain Research Station	CO	3513 m	758 mm	0C/32F	S-Rockies & Colorado Plateau	40.05425	-105.58237	0.58
OAES	Klemme Range Research Station	OK	516 m	670 mm	15C/59F	Southern Plains	35.41059	-99.05879	0.24
OSBS	Ordway-Swisher Biological Station	FL	45 m	1290 mm	20C/68F	Southeast	29.68927	-81.99343	0.92
RMNP	Rocky Mountain National Park, CASTNET	CO	2743 m	520 mm	4C/39.2F	Central Plains	40.27591	-105.54592	0.60
SJER	San Joaquin Experimental Range	CA	368 m	270 mm	17C/62.6F	Pacific Southwest	37.10878	-119.73228	0.76
SOAP	Soaproot Saddle	CA	1160 m	1246 mm	10C/50F	Pacific Southwest	37.03337	-119.26219	0.95
SRER	Santa Rita Experimental Range	AZ	983 m	290 mm	20C/68F	Desert Southwest	31.91068	-110.83549	0.51
TALL	Talladega National Forest	AL	135 m	1350 mm	17C/62.6F	Ozarks Complex	32.95046	-87.39327	1.62
TOOL	Toolik	AK	843 m	331 mm	-4C/24.8F	Tundra	68.66109	-149.37047	0.93
UKFS	The University of Kansas Field Station	KS	335 m	870 mm	12C/53.6F	Prairie Peninsula	39.04043	-95.19215	2.36
WREF	Wind River Experimental Forest	WA	407 m	2530 mm	8C/46.4F	Pacific Northwest	45.82049	-121.95191	1.32
YELL	Yellowstone Northern Range (Frog Rock)	WY	2116 m	509 mm	0C/32F	Northern Rockies	44.95348	-110.53914	0.77

Site characteristics including site abbreviation (Site ID), mean annual precipitation (MAP), mean annual temperature (MAT), latitude, longitude (WGS 84) and mean leaf area index (LAI).

Supplementary Table 2: Linear regression models between taxonomic and spectral distances among plots per site.

Site ID	n	r^2	estimate (*b*)	*P* (sign.)	statistic (*t*)	*df*
ABBY	19	0.34	0.61	<0.001 (***)	35.34	341
BARR	18	0.01	0.41	<0.001 (***)	20.99	305
BART	20	0.42	0.61	<0.001 (***)	59.49	379
BONA	11	0.50	0.67	<0.001 (***)	24.57	109
CLBJ	14	0.36	0.54	<0.001 (***)	26.14	181
DEJU	28	0.22	0.49	<0.001 (***)	53.91	755
DELA	20	0.22	0.51	<0.001 (***)	38.60	379
DSNY	20	0.13	0.49	<0.001 (***)	33.37	379
GRSM	6	0.50	0.51	<0.001 (***)	10.82	29
GUAN	22	0.13	0.36	<0.001 (***)	36.63	461
HARV	21	0.23	0.55	<0.001 (***)	42.41	419
HEAL	30	0.28	0.42	<0.001 (***)	50.86	869
JORN	20	0.43	0.51	<0.001 (***)	38.95	379
KONZ	28	0.15	0.39	<0.001 (***)	46.26	755
LAJA	4	0.38	0.76	<0.001 (***)	6.19	11
LENO	28	0.14	0.41	<0.001 (***)	59.55	755
MLBS	20	0.13	0.46	<0.001 (***)	34.15	379
MOAB	27	0.34	0.36	<0.001 (***)	40.00	701
NIWO	23	0.39	0.54	<0.001 (***)	45.26	505
OAES	32	0.30	0.57	<0.001 (***)	79.29	991
OSBS	11	0.36	0.47	<0.001 (***)	20.87	109
RMNP	18	0.27	0.49	<0.001 (***)	32.04	305
SJER	22	0.08	0.42	<0.001 (***)	35.23	461
SOAP	9	0.20	0.53	<0.001 (***)	14.44	71
SRER	25	0.05	0.31	<0.001 (***)	28.81	599
TALL	22	0.17	0.37	<0.001 (***)	30.77	461
TOOL	26	0.07	0.70	<0.001 (***)	40.79	649
UKFS	24	0.26	0.47	<0.001 (***)	32.94	551
WREF	18	-0.04	0.64	<0.001 (***)	30.65	305
YELL	25	0.12	0.40	<0.001 (***)	39.08	599

Significance was assessed using two-sided t-tests. The number of observations per site (n), proportion of variance explained (r^2), estimates of the slope (*b*), *P*-values and significance levels (*** $P \leq 0.001$, ** $P \leq 0.01$, * $P \leq 0.05$), test statistics (*t*) and degrees of freedom (*df*) are indicated. For site abbreviations see Supplementary Table 1.

Supplementary Table 3: Covariance between plot-wise ordinations of plant species inventories and mean spectra per site.

Site ID	n	*RV*	*P (*sign)
ABBY	19	0.63	0.001 (***)
BARR	18	0.31	0.048 (*)
BART	20	0.59	0.001 (***)
BONA	11	0.70	0.004 (**)
CLBJ	14	0.66	0.001 (***)
DEJU	28	0.22	0.027 (*)
DELA	20	0.34	0.012 (*)
DSNY	20	0.27	0.073
GRSM	6	0.64	0.021 (*)
GUAN	22	0.30	0.018 (*)
HARV	21	0.41	0.002 (**)
HEAL	30	0.52	0.001 (***)
JORN	20	0.38	0.003 (**)
KONZ	28	0.19	0.146
LAJA	4	0.38	0.918
LENO	28	0.24	0.052
MLBS	20	0.44	0.003 (**)
MOAB	27	0.62	0.001 (***)
NIWO	23	0.66	0.001 (***)
OAES	32	0.42	0.001 (***)
OSBS	11	0.53	0.013 (*)
RMNP	18	0.49	0.004 (**)
SJER	22	0.16	0.523
SOAP	9	0.29	0.424
SRER	25	0.29	0.007 (**)
TALL	22	0.30	0.009 (**)
TOOL	26	0.29	0.004 (**)
UKFS	24	0.71	0.001 (***)
WREF	18	0.25	0.088
YELL	25	0.30	0.008 (**)

For each site, the degree of correspondence between ordinations of plant inventories and mean spectra per plot is given by the coefficient of association (*RV*)[50]. Number of observations per site (n), *P*-values and significance levels (*** $P \leq 0.001$, ** $P \leq 0.01$, * $P \leq 0.05$) are averages of 999 Monte-Carlo simulations. For site abbreviations see Supplementary Table 1.

Supplementary Table 4: Best mixed effects model predicting plant species richness.

Term	*statistic (F, df)*	*P* (sign)
Intercept	383.35 (1, 545)	<0.001 (***)
SDalpha	9.23 (1, 545)	0.003 (**)
Vegtype	6.48 (2, 545)	0.002 (**)
Latitude	4.15 (1, 545)	0.042 (*)
meanLAI	12.79 (1, 545)	<0.001 (***)
Elevation	0.01 (1, 24)	0.907
Temperature	0.06 (1, 24)	0.805
Precipitation	0.58 (1, 24)	0.455
Vegtype:Latitude	6.35 (2, 545)	0.002 (**)
Vegtype:Elevation	21.82 (2, 545)	<0.001 (***)
Vegtype:Temperature	2.55 (2, 545)	0.079 (•)
Vegtype:Precipitation	4.98 (2, 545)	0.007 (**)
Latitude:Precipitation	0.72 (1, 545)	0.396
Elevation:Precipitation	5.18 (1, 24)	0.032
Temperature:Precipitation	16.35 (1, 24)	<0.001 (***)

The terms included in the model are spectral alpha-diversity (SDalpha), vegetation type (Vegtype: forest, grassland, shrubland), latitude, mean leaf area index (meanLAI), elevation, mean annual temperature and mean annual precipitation. Analysis-of-variance (ANOVA) results showing the test statistic (two-sided *F*-test), degrees of freedom (*df*), *P*-values and significance levels (*** $P \leq 0.001$, ** $P \leq 0.01$, * $P \leq 0.05$, • $P \leq 0.1$).

Supplementary Table 5: Best mixed effects model predicting the Shannon index of plant diversity.

Term	statistic (*F, df*)	*P* (sign)
Intercept	497.79 (1, 544)	<0.001 (***)
SDalpha	0.72 (1, 544)	0.398
Vegtype	36.4 (2, 544)	<0.001 (***)
Latitude	0.99 (1, 544)	0.320
meanLAI	0.02 (1, 544)	0.891
Elevation	0.14 (1, 26)	0.709
Temperature	0.1 (1, 26)	0.758
Precipitation	0.74 (1, 26)	0.397
SDalpha:Latitude	0.01 (1, 544)	0.915
SDalpha:Temperature	6.16 (1, 544)	0.013 (*)
SDalpha:Precipitation	37.05 (1, 544)	<0.001 (***)
Vegtype:Elevation	7.32 (2, 544)	0.001 (***)
Vegtype:Temperature	0.07 (2, 544)	0.931
Vegtype:Precipitation	6.04 (2, 544)	0.003 (**)
meanLAI:Elevation	3.45 (1, 544)	0.064 (•)

The terms included in the model are spectral alpha-diversity (SDalpha), vegetation type (Vegtype: forest, grassland, shrubland), latitude, mean leaf area index (meanLAI), elevation, mean annual temperature and mean annual precipitation. Analysis-of-variance (ANOVA) results showing the test statistic (two-sided *F*-test), degrees of freedom (*df*), *P*-values and significance levels (*** $P \leq 0.001$, ** $P \leq 0.01$, * $P \leq 0.05$, • $P \leq 0.1$).

Supplementary Table 6: Best mixed effects model predicting plant phylogenetic diversity.

Term	statistic (*F, df*)	*P* (sign)
Intercept	1282.07 (1, 535)	<0.001 (***)
SDalpha	34.77 (1, 535)	<0.001 (***)
Vegtype	2.28 (2, 535)	0.104
Latitude	6.14 (1, 535)	0.014 (*)
meanLAI	7.08 (1, 535)	0.008 (**)
Elevation	0.02 (1, 24)	0.883
Temperature	0.41 (1, 24)	0.528
Precipitation	3.64 (1, 24)	0.069 (•)
SDalpha:Vegtype	5.52 (2, 535)	0.004 (**)
SDalpha:Latitude	3.78 (1, 535)	0.052 (•)
SDalpha:meanLAI	2.76 (1, 535)	0.097 (•)
SDalpha:Elevation	5.24 (1, 535)	0.023 (*)
SDalpha:Temperature	1.40 (1, 535)	0.237
SDalpha:Precipitation	19.82 (1, 535)	<0.001 (***)
Vegtype:Latitude	1.63 (2, 535)	0.196
Vegtype:meanLAI	3.56 (2, 535)	0.029 (*)
Vegtype:Elevation	12.86 (2, 535)	<0.001 (***)
Latitude:meanLAI	1.13 (1, 535)	0.289
Latitude:Temperature	3.38 (1, 535)	0.067 (•)
meanLAI:Elevation	0.003 (1, 535)	0.956
meanLAI:Temperature	4.96 (1, 535)	0.026 (*)
meanLAI:Precipitation	0.16 (1, 535)	0.690
Elevation:Precipitation	0.06 (1, 24)	0.803
Temperature:Precipitation	0.07 (1, 24)	0.793

The terms included in the model are spectral alpha-diversity (SDalpha), vegetation type (Vegtype: forest, grassland, shrubland), latitude, mean leaf area index (meanLAI), elevation, mean annual temperature and mean annual precipitation. As phylogenetic diversity measure, we used phylogenetic species evenness (PSE). Analysis-of-variance (ANOVA) results showing the test statistic (two-sided *F*-test), degrees of freedom (*df*), *P*-values and significance levels (*** $P \leq 0.001$, ** $P \leq 0.01$, * $P \leq 0.05$, • $P \leq 0.1$).

Supplementary References

50 Heo, M. & Gabriel, K.R. A permutation test of association between configurations by means of the rv coefficient. *Communications in Statistics - Simulation and Computation* **27**, 843-856, doi:10.1080/03610919808813512 (1997).
