# Supplementary material for: Plant beta-diversity across biomes captured by imaging spectroscopy
Source: Nat Commun. 2022 May 19;13:2767. doi: 10.1038/s41467-022-30369-6 (PMC9120498; doi:10.1038/s41467-022-30369-6)
Supplement: Supplementary file 3 — Reporting Summary [file 41467_2022_30369_MOESM3_ESM.pdf]

nature portfolio

Corresponding author(s): Anna K. Schweiger

Last updated by author(s): May 5, 2022

# Reporting Summary

Nature Portfolio wishes to improve the reproducibility of the work that we publish. This form provides structure for consistency and transparency in reporting. For further information on Nature Portfolio policies, see our Editorial Policies and the Editorial Policy Checklist.

## Statistics

For all statistical analyses, confirm that the following items are present in the figure legend, table legend, main text, or Methods section.

| n/a | Confirmed | |
|---|---|---|
| ☐ | ☒ | The exact sample size (*n*) for each experimental group/condition, given as a discrete number and unit of measurement |
| ☐ | ☒ | A statement on whether measurements were taken from distinct samples or whether the same sample was measured repeatedly |
| ☐ | ☒ | The statistical test(s) used AND whether they are one- or two-sided<br>*Only common tests should be described solely by name; describe more complex techniques in the Methods section.* |
| ☐ | ☒ | A description of all covariates tested |
| ☐ | ☒ | A description of any assumptions or corrections, such as tests of normality and adjustment for multiple comparisons |
| ☐ | ☒ | A full description of the statistical parameters including central tendency (e.g. means) or other basic estimates (e.g. regression coefficient) AND variation (e.g. standard deviation) or associated estimates of uncertainty (e.g. confidence intervals) |
| ☐ | ☒ | For null hypothesis testing, the test statistic (e.g. *F*, *t*, *r*) with confidence intervals, effect sizes, degrees of freedom and *P* value noted<br>*Give P values as exact values whenever suitable.* |
| ☒ | ☐ | For Bayesian analysis, information on the choice of priors and Markov chain Monte Carlo settings |
| ☒ | ☐ | For hierarchical and complex designs, identification of the appropriate level for tests and full reporting of outcomes |
| ☐ | ☒ | Estimates of effect sizes (e.g. Cohen's *d*, Pearson's *r*), indicating how they were calculated |

*Our web collection on statistics for biologists contains articles on many of the points above.*

## Software and code

Policy information about availability of computer code

| | |
|---|---|
| Data collection | No data was collected for this study. |
| Data analysis | For data analysis we used R version 3.6.2., and the R packages vegan version 2.5-7, V.PhyloMaker version 0.1.0, picante version 1.8.2, ape 5.0, ade4 version 1.7-16, nlme version 3.1-152; the code for calculating crown area of woody plants as seen from above can be found here: https://github.com/annakat/NEON_crown_area (https://doi.org/10.5281/zenodo.6383923); the code for spectra diversity calculations can be found here: https://github.com/elaliberte/specdiv (https://doi.org/10.5281/zenodo.6385476). |

For manuscripts utilizing custom algorithms or software that are central to the research but not yet described in published literature, software must be made available to editors and reviewers. We strongly encourage code deposition in a community repository (e.g. GitHub). See the Nature Portfolio guidelines for submitting code & software for further information.

## Data

Policy information about availability of data

All manuscripts must include a data availability statement. This statement should provide the following information, where applicable:

- Accession codes, unique identifiers, or web links for publicly available datasets
- A description of any restrictions on data availability
- For clinical datasets or third party data, please ensure that the statement adheres to our policy

All data used this analysis are available from the National Ecological Observatory Network (NEON): https://doi.org/10.48443/qeae-3x15, https://doi.org/10.48443/4e85-cr14, https://doi.org/10.48443/abge-r811, https://doi.org/10.48443/e3qn-xw47, https://doi.org/10.48443/h2rb-pj34.

# Field-specific reporting

Please select the one below that is the best fit for your research. If you are not sure, read the appropriate sections before making your selection.

☐ Life sciences ☐ Behavioural & social sciences ☒ Ecological, evolutionary & environmental sciences

For a reference copy of the document with all sections, see nature.com/documents/nr-reporting-summary-flat.pdf

# Ecological, evolutionary & environmental sciences study design

All studies must disclose on these points even when the disclosure is negative.

| | |
|---|---|
| Study description | We used plant inventories and remote sensing data collected by the National Ecological Observatory Network (NEON) to test the degree to which spectral diversity predicts plant diversity at local and regional scales. We used data from 30 NEON sites and 4-32 plots per site. Plots were distributed to cover all vegetation types per site (nested design). |
| Research sample | Plant inventories were collected by NEON field personnel, and airborne image data by the NEON Airborne Biodiversity Observatory (AOP) across all sites in the United States. Data are available from the NEON data repository: https://doi.org/10.48443/qeae-3x15, https://doi.org/10.48443/4e85-cr14, https://doi.org/10.48443/abge-r811, https://doi.org/10.48443/e3qn-xw47, https://doi.org/10.48443/h2rb-pj34 |
| Sampling strategy | The sampling strategy for plant inventories can be found under "Collection and Processing" at https://data.neonscience.org/data-products/DP1.10058.001 and https://data.neonscience.org/data-products/DP1.10098.001. The sampling strategy follows a nested design; inventories were collected in subplots per plot until no additional species were encountered. For details on the NEON's imaging spectrometer (NIS) on the AOP please see https://www.neonscience.org/data-collection/imaging-spectrometer. |
| Data collection | All data used in this study were collected by NEON staff, see "Collection and Processing" https://data.neonscience.org/data-products/DP1.10058.001, and https://www.neonscience.org/data-collection/imaging-spectrometer. Species inventories were collected in the field, imaging spectroscopy data were collected by the NEON imaging spectrometer (NIS) is a passive remote sensing instrument that measures reflected solar energy from 380 to 2500 nm in 5 nm bands. |
| Timing and spatial scale | Spectral image data were collected between April 1 and October 7 2018. Spectral images tiles used in this study measured 1km x 1km. Vegetation inventories were collected at peak growing season between 2016 and 2019. Vegetation plots measure 20m x 20m. NEON collects data across all sampling sites in the US using a rotating scheme, because for logistical reasons not all sites can be assessed every year. We used the plant inventories conducted closest in time to spectral image acquisition. |
| Data exclusions | We excluded plots that were dominated (>50%) by shade and non-photosynthetically active vegetation. |
| Reproducibility | We used different thresholds for the exclusion of shaded and non-photosynthetically active vegetation (see Methods), but this did not substantially affect our results. No true replicates were used in this study. |
| Randomization | For co-variance analysis we used averages of 999 Monte-Carlo simulations. For calculating phylogenetic diversity we generated 100 phylogenies using V.phylomaker in R, based on the phylogeny released by Smith and Brown (2018), with new tips bound to randomly selected nodes at and below the genus level. |
| Blinding | No blinding was used since our study did not involve animals or human research participants. |

Did the study involve field work? ☐ Yes ☒ No

# Reporting for specific materials, systems and methods

We require information from authors about some types of materials, experimental systems and methods used in many studies. Here, indicate whether each material, system or method listed is relevant to your study. If you are not sure if a list item applies to your research, read the appropriate section before selecting a response.

## Materials & experimental systems

| n/a | Involved in the study | |
|---|---|---|
| ☒ | ☐ | Antibodies |
| ☒ | ☐ | Eukaryotic cell lines |
| ☒ | ☐ | Palaeontology and archaeology |
| ☒ | ☐ | Animals and other organisms |
| ☒ | ☐ | Human research participants |
| ☒ | ☐ | Clinical data |
| ☒ | ☐ | Dual use research of concern |

## Methods

| n/a | Involved in the study | |
|---|---|---|
| ☒ | ☐ | ChIP-seq |
| ☒ | ☐ | Flow cytometry |
| ☒ | ☐ | MRI-based neuroimaging |
